# Comparison of four different reduction methods for anterior dislocation of the shoulder

**DOI:** 10.1186/s13018-015-0226-4

**Published:** 2015-05-28

**Authors:** Olcay Guler, Safak Ekinci, Faruk Akyildiz, Uzeyir Tirmik, Selami Cakmak, Akin Ugras, Ahmet Piskin, Mahir Mahirogullari

**Affiliations:** Orthopedics and Traumatology Department, Medical Faculty, Medipol University, Atatürk Bulvarı. No:27 Unkapanı, 34083 Fatih, Istanbul Turkey; Orthopedics and Traumatology Department, Agrı Military Hospital, Agrı, Turkey; Orthopedics and Traumatology Department, Malatya Military Hospital, Malatya, Turkey; Orthopedics and Traumatology Department, Etimesgut Military Hospital, Ankara, Turkey; Orthopedics and Traumatology Department, Gulhane Military Medical Academy Haydarpasa Training Hospital, Istanbul, Turkey; Orthopedics and Traumatology Department, Medical Faculty, Ondokuz Mayıs University, Samsun, Turkey

**Keywords:** Shoulder, Anterior shoulder dislocation, Closed reduction

## Abstract

**Background:**

Shoulder dislocations account for almost 50 % of all major joint dislocations and are mainly anterior.

**Objective:**

The aim is a comparative retrospective study of different reduction maneuvers without anesthesia to reduce the dislocated shoulder.

**Methods:**

Patients were treated with different reduction maneuvers, including various forms of traction and external rotation, in the emergency departments of four training hospitals between 2009 and 2012. Each of the four hospitals had different treatment protocols for reduction and applying one of four maneuvers: Spaso, Chair, Kocher, and Matsen methods. Thirty-nine patients were treated by the Spaso method, 47 by the Chair reduction method, 40 by the Kocher method, and 27 patients by Matsen’s traction-countertraction method. All patients’ demographic data were recorded. Dislocation number, reduction time, time interval between dislocation and reduction, and associated complications, pre- and post-reduction period, were recorded prospectively. No anesthetic method was used for the reduction.

**Results:**

All of the methods used included traction and some external rotation. The Chair method had the shortest reduction time. All surgeons involved in the study agreed that the Kocher and Matsen methods needed more force for the reduction. Patients could contract their muscles because of the pain in these two methods. The Spaso method includes flexion of the shoulder and blocks muscle contraction somewhat. The Chair method was found to be the easiest because the patients could not contract their muscles while sitting on a chair with the affected arm at their side.

**Conclusions:**

We suggest that the Chair method is an effective and fast reduction maneuver that may be an alternative for the treatment of anterior shoulder dislocations. Further prospective studies with larger sample size are needed to compare safety of different reduction techniques.

## Background

Shoulder dislocations account for nearly 50 % of all major joint dislocations presenting to emergency departments [1]. Very often, shoulder dislocations are anterior (90–98 %) and occur due to trauma [2]. The primary anterior dislocation incidence is estimated to be around 12.3 per 100,000 people [3]. Many reduction methods have been described in the literature [1]. The methods include different reduction maneuvers. However, few studies have compared the efficacy, reliability, and safety of the various techniques [1–3]. As a result, deciding which technique to use is seldom based on objective criteria. Which method is superior is also unclear. An “ideal” reduction method would be effective, rapid, and as painless as possible for patients and should not cause iatrogenic complications.

The aim of this study was to compare the clinical outcome, primarily reduction time and pain, of four different reduction maneuvers to reduce the dislocated shoulder, which were all performed without anesthesia.

## Patients and methods

### Study design and population

In total, 162 patients who were treated with any of four reduction maneuvers, with different forms of traction and external rotation (Spaso, Chair, Kocher, and Matsen methods) in the emergency units of four hospitals between 2009 and 2012, were retrospectively included in the study. Each of the four hospitals had different treatment protocols for reduction and applying one of the four maneuvers.

Patients aged 18 years or over who had acute traumatic anterior shoulder dislocation, were cooperative, and could communicate were included. Patients with hemodynamic instability (two patients), polytrauma (one patient), Ideberg type 2–5 glenoid fracture associated with dislocation (one patient) [4], and recurrent dislocation with a history of reduction under sedative/anxiolytic/analgesic/muscle relaxant (two patients) and those who wanted sedation prior to the reduction maneuver (three patients) were excluded. Thus, nine patients were excluded from the study, leaving 153 patients (36 females, 127 males) for analysis.

Anterior shoulder dislocation was diagnosed by physical examination and radiography. Conventional antero-posterior and trans-scapular view plain radiographs were taken pre- and post-reduction. Each of the four reduction maneuvers was performed by one of four physicians who were in the third year of residencies in orthopedic surgery.

This retrospective study was approved by the Institutional Ethics Committee of Medipol University and was conducted in accordance with the latest version of the Helsinki Declaration. All patients were informed about the study and signed an informed consent form.

### Reduction techniques

#### Kocher method

**Fig. 1:**
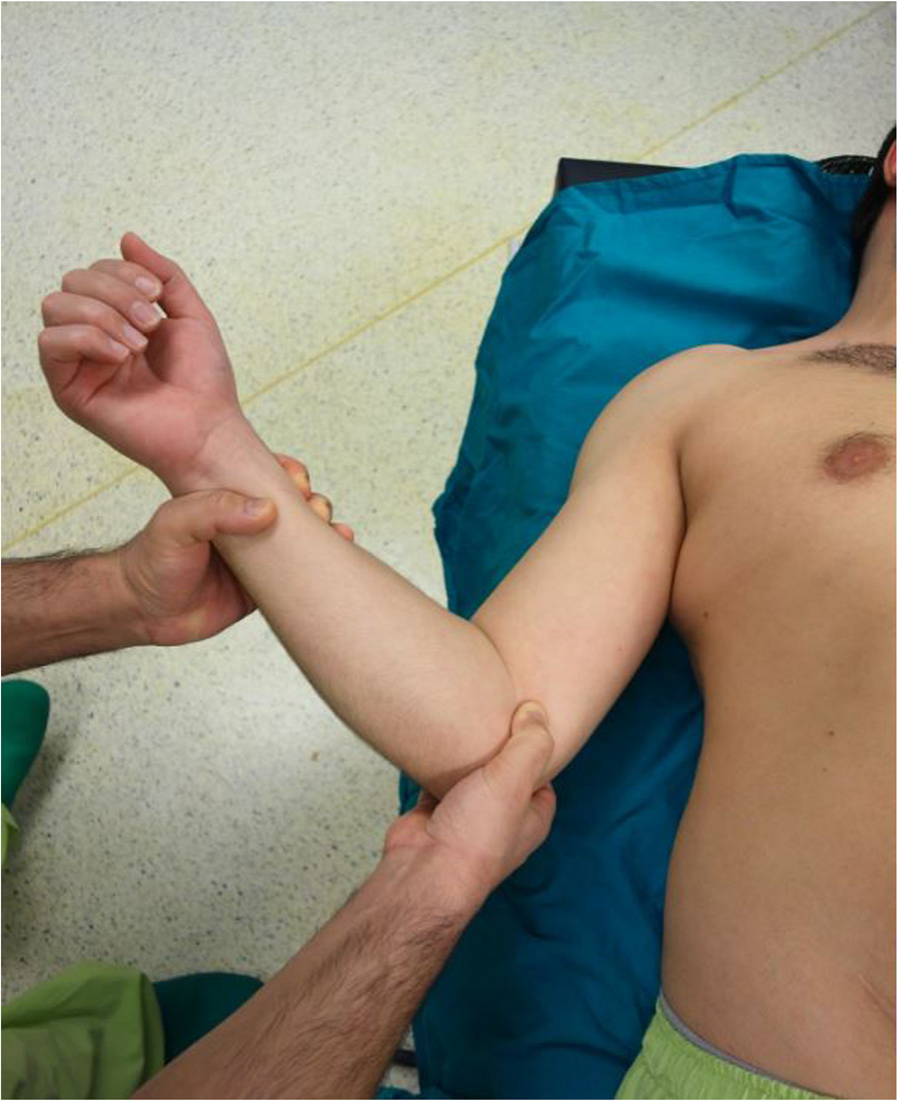
Position of the patient in Kocher’s technique

Originally described by Kocher in 1870, Kocher’s method does not involve traction [5]. Kocher’s technique was performed and modified by Watson-Jones [6]. In this technique, the patient was placed supine on the examining table with the physician standing at his/her side (Fig. 1).

The patient bends the affected arm at 90° at the elbow and adducts it against the body to allow the wrist and the point of the elbow to be grasped by the physician. The shoulder is slowly rotated externally between 70° and 85° until resistance is felt. The externally rotated upper arm is lifted in the sagittal plane as forward as possible, and the shoulder is internally rotated to bring the patient’s hand towards the opposite shoulder. The humeral head should now slip back into the glenoid fossa with pain eliminated during this process.

#### Spaso method

**Fig. 2:**
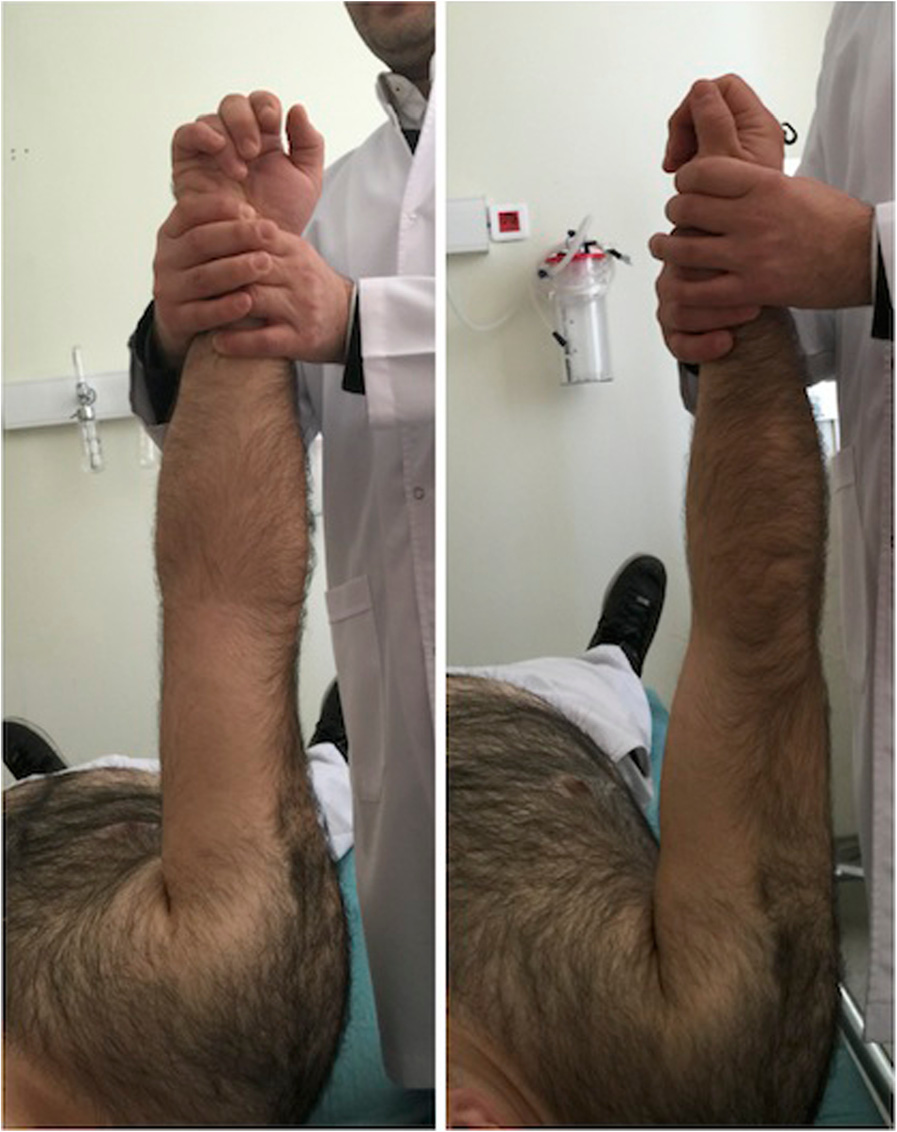
Spaso method of reduction for shoulder dislocation

The patient is placed in the supine position; the affected arm is grasped around the wrist or the distal forearm and gently lifted vertically, applying gentle traction. While maintaining vertical traction, the shoulder is slightly rotated externally. A clunk is heard and/or felt when the reduction is completed (Fig. 2).

Reduction will usually occur after a few minutes of gentle traction. If difficulty is experienced, it may be helpful to use one hand to palpate the head of the humerus and gently push it to assist the reduction, while maintaining traction with the other hand [7–9].

#### Chair method

**Fig. 3:**
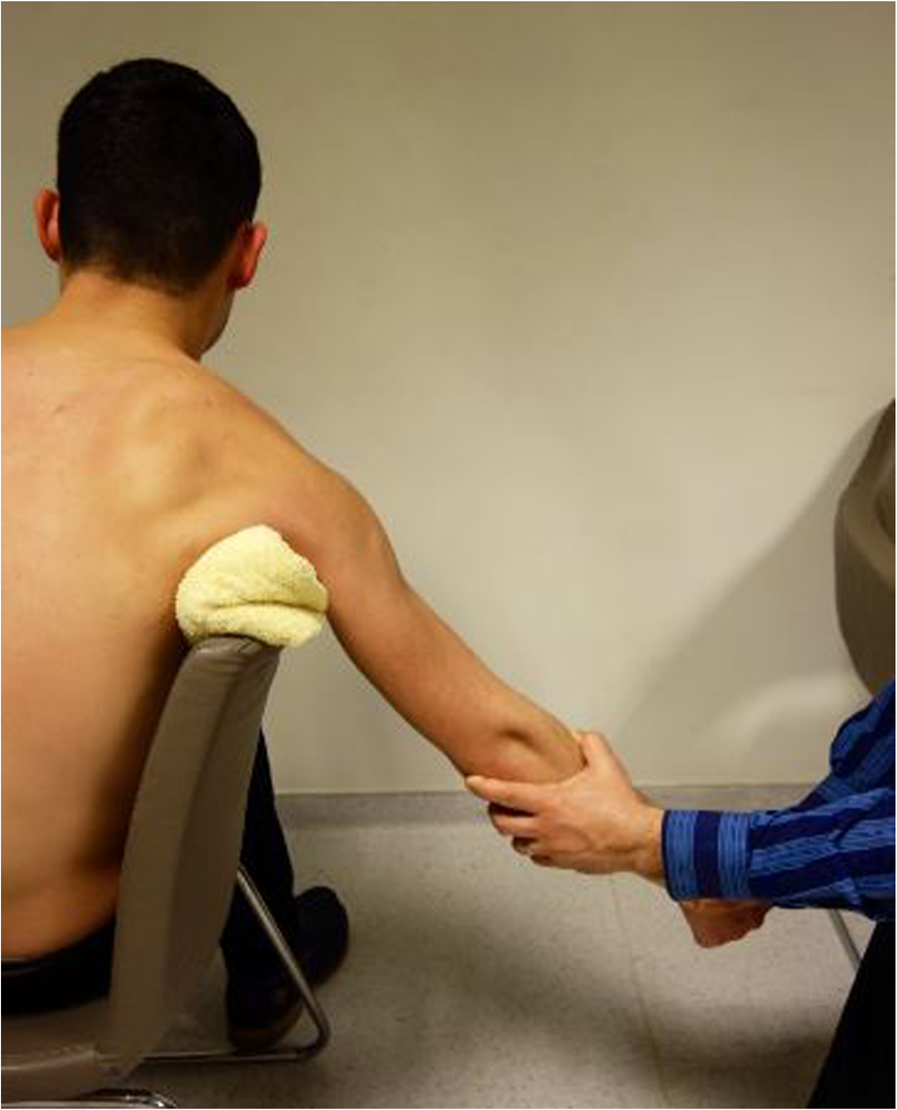
Chair method of reduction for shoulder dislocation

The patient is asked to sit in a stable chair sideways using the backrest of the chair as a fulcrum in the axilla. If the backrest of the chair is not well-padded, it is supported by a folded bed sheet or small, stiff pillow. Thus, the risks of an axillary nerve injury or iatrogenic fracture are minimized. The dislocated arm is allowed to hang over the backrest of the chair. The physician squats down behind the chair, holds the patient’s elbow with the left hand for a right shoulder dislocation, and induces the patient’s arm to gently flex at the elbow (Fig. 3).

The physician’s other hand holds the patient’s right hand without performing a maneuver. The patient is asked and encouraged to relax and be calm; traction is applied slowly by the left hand of the physician, and reduction occurs at this stage. If the humeral head is stacked at the inferior margin of the glenoid, a slight amount of external rotation can be applied by the right hand of the physician [10, 11].

#### Matsen’s traction-countertraction method

**Fig. 4:**
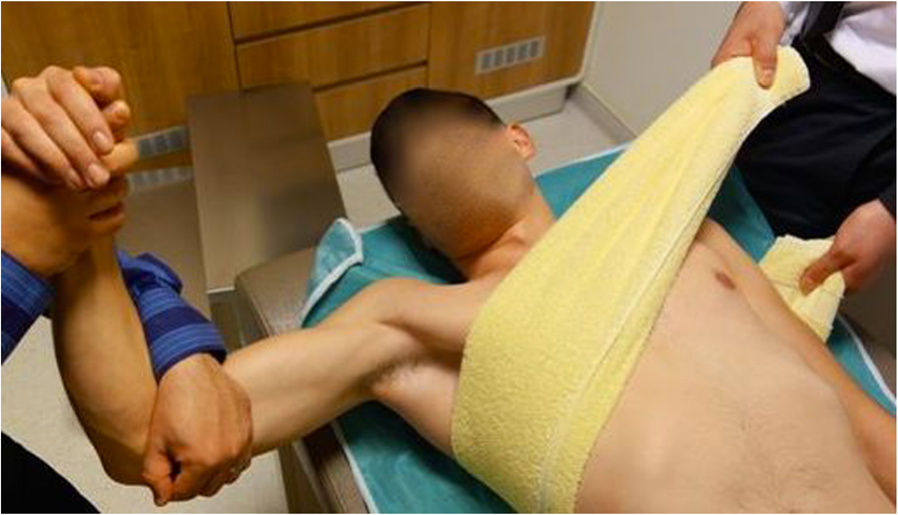
Matsen’s traction-countertraction method of reduction for shoulder dislocation

The patient is placed on his/her back with a sheet around the chest and also around the assistant’s waist for countertraction. The physician stands on the side of the dislocated shoulder near the patient’s waist with the elbow of the dislocated shoulder bent to 90°. A second sheet, tied loosely around the physician’s waist and looped over the patient’s forearm, provides traction while the physician leans back against the sheet while grasping the forearm. Traction is applied to the arm with the shoulder in abduction, and the assistant applies firm countertraction to the body using a folded sheet (Fig. 4) [5].

### Follow-up procedures

In patients who failed the first reduction maneuver, the reduction was achieved with one of the other reduction methods. Failed reduction maneuvers were recorded as failures, while successful second reductions were recorded as successful. All patients were immobilized in internal rotation with a shoulder/arm sling for 3 weeks after the reduction. After 3 weeks, the patients underwent rehabilitation.

Demographic data, the number of dislocations, cause of injury, dislocation side, the dominant limb, pre- and post-reduction neurovascular examination findings, reduction time, duration of emergency unit stay, the presence of a tuberculum majus fracture, and complications during pre- and post-reduction period were recorded for all patients.

The visual analog scale (VAS) scoring system was used to assess intra- and post-reduction degree of pain, which was scored from 0 (no pain) to 10 (extremely severe). The same orthopedic surgeon who had performed the reduction asked the patients their level of pain and marked the answer on the VAS scoring system.

### Statistical analyses

Study data were summarized using descriptive statistics (mean, standard deviation, frequency, percentage). Comparison of categorical variables between reduction methods was performed using the *χ*^2^ test or Fisher’s exact test. Analysis of variance (ANOVA) was used to compare continuous data for the four reduction methods, followed by Tukey’s test for post hoc analyses. The level of statistical significance was set at *p* < 0.05. The statistical power to determine the probability of detecting 1.0 difference in the VAS score between four reduction techniques with 1.5 standard deviation and type I error of 0.05 changes between 0.82 and 0.98 for a sample size of 27 to 57 per group, respectively. The power calculation for ANOVA was performed in www.statstodo.com (Statstodo Trading Pty Ltd). For statistical analyses, the MedCalc software (ver. 12.7.7; MedCalc Software bvba, Ostend, Belgium; http://www.medcalc.org; 2013) was used.

## Results

In total, 153 patients (127 males, 26 females; mean age, 36.8 years; age range, 19–52 years) were included in the study. Of them, 39 patients (32 males, 7 females) were treated by the Spaso method, 47 patients (42 males, 5 females) by the Chair reduction method, 40 patients (31 males, 9 females) by Kocher’s method, and 27 patients (20 males, 7 females) by Matsen’s traction-countertraction method. Age and gender distributions of patients were similar between reduction methods (Table 1). Most patients were right-handed in all groups, and dislocation was in the dominant arm in 60 % of patients without significant difference between reduction methods (Table 1). Body mass index of patients was also similar in all groups (Table 1).

**Table 1:** Clinical data of shoulder dislocation patients in whom Chair, Matsen, Spaso, or Kocher reduction methods were applied

	Chair method	Kocher method	Spaso method	Matsen method	*p* value
	*n* = 47	*n* = 40	*n* = 39	*n* = 27	
Age (years)					
Female	41.4 ± 8.1	39.7 ± 11.4	42.1 ± 9.3	37.9 ± 10.4	0.98^a^
Male	35.7 ± 12.4	32.4 ± 9.6	37.9 ± 13.3	34.6 ± 9.4	
Gender					
Female	5 (10.6 %)	9 (22.5 %)	7 (17.9 %)	7 (25.9 %)	0.3379^b^
Male	42 (89.4 %)	31 (77.5 %)	32 (82.1 %)	20 (74.1 %)	
Dominant arm					
Right	40 (85.1 %)	34 (85 %)	35 (89.7 %)	22 (81.4 %)	0.8165^c^
Left	7 (14.9 %)	6 (15 %)	4 (10.3 %)	5 (18.6 %)	
Dislocation in dominant arm	29 (61.7 %)	25 (62.5 %)	22 (56.4 %)	16 (59.2 %)	0.9509^b^
Body mass index (kg/m^2^)	29.3 ± 6.1	30.1 ± 5.4	28.1 ± 6.4	27.9 ± 7.1	0.825^c^
Number of dislocation					
First dislocation	28 (59.5 %)	23 (57.5 %)	24 (61.5 %)	18 (66.7 %)	0.8939^b^
Recurrent	19 (40.5 %)	17 (42.5 %)	15 (38.5 %)	9 (33.3 %)	
Side					
Right	26 (55.3 %)	21 (52.5 %)	22 (56.4 %)	15 (55.5 %)	0.9871^b^
Left	21 (44.7 %)	19 (47.5 %)	17 (43.6 %)	12 (44.5 %)	
Reason for dislocation					
Sport trauma	24 (51 %)	20 (50 %)	20 (51.2 %)	15 (55.5 %)	0.9668^c^
Fall	21 (44.6 %)	17 (42.5 %)	18 (46.1 %)	11 (40.7 %)	
Traffic accidents	2 (4.4 %)	3 (7.5 %)	1 (2.7 %)	1 (3.8 %)	
Tuberculus majus fracture	1 (2.1 %)	2 (5 %)	1 (2.5 %)	1 (3.7 %)	0.8848^c^
Pre-reduction neurologic deficit	5 (10.6 %)	3 (7.5 %)	4 (10.2 %)	3 (11.1 %)	0.9527^c^
Success rate	46 (97.8 %)	39 (97.5 %)	37 (94.8 %)	25 (92.5 %)	0.6509^c^

^a^ANOVA test

^b^Chi-square test

^c^Fisher’s exact test

No anesthetic or analgesic method was used during any reduction. Six patients who failed the initial reduction technique were treated successfully by one of the other reduction techniques. A patient who failed the Kocher technique underwent the Chair method, another who failed the Chair underwent the Kocher method, two patients who failed the Matsen method underwent the Spaso method, and two patients who failed the Spaso underwent the Matsen method.

The number, side of dislocation, reason for dislocation, presence of tuberculum majus fracture, and pre-reduction neurological deficit ratio were similar among the four techniques (Table 1).

All four reduction techniques provided high success rates with no statistically significant difference among them (46/47, 39/40, 37/39, and 25/27, for Chair, Kocher, Spaso, and Matsen, respectively; *p* = 0.6509; Table 1).

Post-reduction radiographies revealed no displacement in any patients with tuberculum majus fractures, and fracture union was obtained with conservative treatment. Additionally, no new fracture was seen in post-reduction radiographies. Pre-reduction neurological deficits were improved after reduction in all patients. None of the patients had complications like proximal humeral fractures or neurological injuries.

Reduction time, first dislocation reduction time, and intra-reduction VAS were significantly different among the groups, being lowest in patients who were treated with the Chair method (Table 2).

**Table 2:** Outcome parameters for different reduction techniques against shoulder dislocation

	Chair (C)	Kocher (K)	Spaso (S)	Matsen (M)	*p* value^a^	*p* values for post hoc comparisons^b^					
	(*n* = 47)	(*n* = 40)	(*n* = 39)	(*n* = 27)		C-K	C-S	C-M	K-S	K-M	S-M
Reduction time (min)	3.0 ± 1.2	4.9 ± 1.4	4.8 ± 1.5	4.7 ± 2.3	0.011	<0.001	<0.001	<0.001	0.8211	0.6587	0.7953
First dislocation reduction time (min)	3.2 ± 1.4	5.3 ± 2.8	5.2 ± 2.2	5.0 ± 2.0	0.005	<0.001	<0.001	<0.001	0.9046	0.7144	0.7227
Recurrent dislocation reduction time (min)	3.1 ± 1.1	4.1 ± 1.3	4.0 ± 1.3	3.9 ± 1.0	0.053	–	–	–	–	–	–
Emergency department time (min)	85.0 ± 15.5	92.0 ± 20.5	96.0 ± 19.5	88.0 ± 22.5	0.74	–	–	–	–	–	–
Intra-reduction VAS	4.0 ± 2.4	6.9 ± 2.6	6.5 ± 2.8	6.8 ± 2.3	0.03	<0.001	<0.001	<0.001	0.5414	0.8727	0.6181
Post-reduction VAS	2.1 ± 1.1	2.6 ± 1.4	2.7 ± 1.5	3.0 ± 1.6	0.543	–	–	–	–	–	–

^a^ANOVA

^b^Tukey test

## Discussion

Currently, no single shoulder reduction method has a 100 % success rate, and no technique has been found to be ideal in every shoulder dislocation situation. An ideal method should be simple, rapid, effective, painless, and free of complications and should facilitate rapid patient disposition [12, 13].

Today, many departments reduce shoulder dislocations either under general anesthetic or with the aid of parenteral analgesia or sedation [11, 14, 15]. However, this requires the use of further staff during the procedure and afterwards to observe the recovery period. Sedation/analgesia has usually been recommended when the procedure has been unsuccessful [15]. Manipulation without sedatives or anesthetic allows rapid patient recovery, reducing the time the patient spends in the department and freeing medical and nursing staff for other tasks [16]. Any additional procedure such as sedation, local analgesics, or anesthesia during shoulder reduction can delay the reduction time and increase the length of stay in the emergency department. Furthermore, the benefits of reduction without sedation can save staff time in the emergency department and facilitate rapid patient disposition [17]. Tezel et al. reported that a longer time was spent in the emergency department after shoulder reduction with suprascapular nerve block and sedation [18]. Other potential complications include residual sedative effects, respiratory complications, cardiovascular complications, permanent brain damage, and even death [19]. Unnecessary sedation should be avoided to reduce the chances of potential complications wherever possible. Chung et al. found a significantly shorter length of stay with the Oxford Chair method versus traditional methods without sedation [20]. In the present study, different reduction maneuvers were performed successfully without analgesics or anesthetics. None of our patients received sedation, anxiolytic treatment, or general anesthesia.

Many previous studies reported that muscle contraction causes difficulty and pain during reduction [21–23]. The muscle contraction is usually caused by increase in pain during traction in patients with limited pain tolerance, which then creates a common problem in reduction [21–23]. The level of pain should be assessed and controlled for an effective shoulder reduction. The VAS is a widely used, validated scale for measuring pain and an effective instrument for surgical investigations. However, studies that measure pain with VAS are limited. In a study comparing Stimson and Milch reduction techniques, Amar et al. recorded VAS pain score as 5.3 and 5.4, respectively [24]. Sayegh et al. reported VAS scores of 1.6, 4.9, and 5.4 with FARES, Hippocratic, and Kocher techniques [25]. In our study, VAS pain score was significantly lowest for the Chair procedure, followed in order by the Kocher, Matsen, and Spaso methods (4.0, 6.9, 6.8, and 6.5, respectively; *p* = 0.03).

Choosing the best technique for the reduction of a shoulder dislocation is often a multifactorial decision. The clinician must weigh the merits of each technique against factors such as sedation risks, availability of assistance, patient anxiety, and comfort level of the operator. Sometimes, more than one technique will be attempted for the successful reduction of the shoulder [26]. Many methods for reduction of anterior shoulder dislocation have been reported, all of which including traction and minimal external rotation. Traction provides sliding the humeral head from the anteroinferior glenoid rim; the humeral head is perched on the edge of the glenoid, and then humeral head rolls on the glenoid rim with external rotation [5]. In terms of reduction maneuvers, the Spaso method looks very simple. Although the Spaso method is associated with some disadvantages like having no mechanism to prevent muscle contraction and need for forward flexion of shoulder, high success rates (67.6–87.5 %) without complications have been reported [8, 9, 27]. In the Matsen traction-countertraction technique, a fully relaxed patient and an assistant for applying strong countertraction are needed [28]. In the Kocher technique modified by Watson-Jones, in spite of need for a relaxed patient and increased risk of proximal humerus fractures, there is no need for countertraction and the success rate reaches to 68–93 % [21, 25, 29, 30]. The Chair technique has the disadvantage of need for a chair but reported a success rate over 90 % [11, 31]. The success rate in our study was 97.8, 97.5, 94.8, and 92.5 % for Chair, Matsen, Spaso, and Kocher techniques, respectively, without significant difference between techniques. However, initial reduction resulted in failure in six patients. Therefore, it should be noted that multiple reduction techniques need to be applied for anterior shoulder dislocations in emergency departments.

All the induction methods used in the present study include some traction and external rotation. All surgeons involved in this study agreed that the Kocher and Matsen methods needed more force to reduce than the other methods. Patients may contract their muscle because of pain with these two methods. The Chair method was found to be the easiest reduction method according to all physicians because the patients could not contract their muscles while sitting on a chair with the affected arm at their side. The duration of reduction varies for each technique, and in comparative studies, no superiority has been demonstrated for any technique [15, 16, 20, 32]. Sayegh et al. reported longer reduction duration in the Kocher technique (mean, 4.3 min) compared to the FARES technique [25]. Amar et al. recorded reduction duration as 8.82 and 4.68 min in Stimson and Milch techniques, respectively [24]. On the other hand, in a comparative study by Chung et al., the mean reduction duration was 3 and 5 min in Chair and Kocher techniques, respectively [20]. In our opinion, each physician has his/her own technique in which they have been trained, and typically uses it. We compared four different reduction techniques; the Chair method was found to be associated with significantly shorter reduction duration. Compared to other methods, the Chair technique causes less pain during reduction, which allows the fastest reduction and the shortest reduction time (3.0 min for the Chair method; 4.9, 4.8, and 4.7 min for Kocher, Spaso, and Matsen, respectively, *p* = 0.011). The main disadvantage of the Chair method is the need for a chair. Finding an appropriate chair can be difficult in certain circumstances. Another drawback is the need for the patient to be conscious and alert. This method cannot be used in non-cooperative patients and in the presence of other injuries preventing the patient from sitting comfortably on a chair.

It is crucial to overcome the muscular resistance of the patient during these maneuvers. If a reduction maneuver is continued persistently against resistance, complications such as excessive pain or fracture (e.g., humoral neck fractures during reduction of anterior shoulder dislocation particularly in osteoporotic bones) and brachial plexus and axillary nerve damage may occur [4, 5, 7, 9, 10, 12–18, 21–23]. Hippocratic and Kocher techniques are commonly associated with axillary nerve injury, humeral shaft and neck fractures, and capsular damage [33]. Pectoralis major rupture has been reported as a rare complication of Kocher’s maneuver [34]. In our study, none of the patients had any such complications, probably due to long-term experience with the same technique of participating emergency clinics.

The main limitations of the study were its retrospective design and low sample size to detect clinically important but small differences between reduction methods, which both prevent reaching a definitive conclusion. Furthermore, each of the four study groups was included from four different hospitals with different clinical practices; thus, the groups were heterogeneous. This study also did not evaluate any possible long-term soft tissue pathologies. Nevertheless, this study presents clinical comparison of multiple reduction techniques for shoulder dislocation.

In conclusion, multiple reduction techniques are available for shoulder dislocation. Physicians working in the emergency departments should become familiar with many techniques for reducing anterior dislocations of the shoulder, because no single method has a 100 % success rate. On the basis of our findings, we suggest that the Chair method is an effective and fast reduction maneuver that may be an alternative for the treatment of anterior shoulder dislocations. Further prospective studies with larger sample size are needed to compare safety of different reduction techniques.
